# The impact of incorporating Bayesian network meta-analysis in cost-effectiveness analysis - a case study of pharmacotherapies for moderate to severe COPD

**DOI:** 10.1186/1478-7547-12-8

**Published:** 2014-03-13

**Authors:** Kristian Thorlund, Zafar Zafari, Eric Druyts, Edward J Mills, Mohsen Sadatsafavi

**Affiliations:** 1 Stanford Prevention Research Center, Stanford University, Stanford, CA, USA; 2 Department of Clinical Epidemiology and Biostatistics, McMaster University, Hamilton, ON, Canada; 3 Collaboration for Outcome Research and Evaluations, Faculty of Pharmaceutical Sciences, University of British Columbia, Vancouver, Canada; 4 Department of Experimental Medicine, University of British Columbia, Vancouver, BC, Canada; 5 Faculty of Medicine, University of Ottawa, Ottawa, ON, Canada; 6 Faculty of Medicine, University of British Columbia, Vancouver, BC, Canada

**Keywords:** Meta-analysis, Multiple treatment comparison, Bayesian analysis, Cost-effectiveness

## Abstract

**Objective:**

To evaluate the impact of using network meta-analysis (NMA) versus pair wise meta-analyses (PMA) for evidence synthesis on key outputs of cost-effectiveness analysis (CEA).

**Methods:**

We conducted Bayesian NMA of randomized clinical trials providing head-to-head and placebo comparisons of the effect of pharmacotherapies on the exacerbation rate in chronic obstructive pulmonary disease (COPD). Separately, the subset of placebo–comparison trials was used in a Bayesian PMA. The pooled rate ratios (RR) were used to populate a decision-analytic model of COPD treatment to predict 10-year outcomes.

**Results:**

Efficacy estimates from the NMA and PMA were similar, but the NMA provided estimates with higher precision. This resulted in similar incremental cost-effectiveness ratios (ICER). Probabilities of being cost-effective at willingness-to-pay thresholds (WTPs) between $25,000 and $100,000 per quality adjusted life year (QALY) varied considerably between the PMA- and NMA-based approaches. The largest difference in the probabilities of being cost-effective was observed at a WTP of approximately $40,000/QALY. At this threshold, with the PMA-based analysis, ICS, LAMA and placebo had a 43%, 30, and 18% probability of being the most cost-effective. By contrast, with the NMA based approach, ICS, LAMA, and placebo had a 56%, 19%, and 21% probability of being cost-effective. For larger WTP thresholds the probability of LAMA being the most cost-effective became higher than that of ICS. Under the PMA-based analyses the cross-over occurred at a WTP threshold between $60,000/QALY-$65,000/QALY, whereas under the NMA-based approach, the cross-over occurred between $85,000/QALY-$90,000/QALY.

**Conclusion:**

Use of NMAs in CEAs is feasible and, as our case study showed, can decrease uncertainty around key cost-effectiveness measures compared with the use of PMAs. The approval process of health technologies in many jurisdictions requires estimates of comparative efficacy and cost-effectiveness. NMAs play an increasingly important role in providing estimates of comparative efficacy. Their use in the CEAs therefore results in methodological consistency and reduced uncertainty.

## Introduction

Network meta-analysis (NMA) (also known as multiple or mixed treatment comparisons) are becoming widely accepted for establishing comparative efficacy between competing health technologies [1-4]. In contrast with conventional pair wise meta-analysis (PMA), NMAs allow for comparisons between interventions that have not been compared head-to-head in randomized clinical trials (RCTs), and offer additional precision by ‘borrowing strength’ from indirect evidence [1,2,5-7]. In medical decision-making, NMAs are commonly used in health technology assessments produced by government agencies or pharmaceutical companies in connection with technology approval submissions [8-10]. In this context, NMAs can provide reliable and consistent evidence on the efficacy and safety of the considered interventions. The contemporary technology approval process in many jurisdictions is informed by evaluating comparative efficacy as well as cost-effectiveness analysis (CEA) comparing the new technology with the alternative choices. NMAs are increasingly popular frameworks for synthesizing evidence on comparative efficacy [2,3]. Despite the merits of NMAs, it is still common that evidence synthesis for the CEA is based on conventional PMA meta-analysis. While some integration of NMAs and CEAs are beginning to take place in commercially prepared health technology assessment (HTA) reports, we are not aware of any published applications intended to inform decision-making.

In addition, it is well accepted that CEAs should be comprehensive [11]. That is, the analysis should include all available treatment options; and the evidence synthesis should be based on all the available evidence [12]. A CEA based on PMA meta-analyses may however fall short in these two aims. First, evidence on comparative efficacy and safety may not be available for all treatments via PMA meta-analysis because not all options have been compared head-to-head or with a common control intervention. Second, when more than two options are compared, the evidence synthesis for a PMA is often based on taking one technology as the ‘reference’ and looking for comparative studies of other technologies with that reference. In this vein, head-to-head comparisons between the considered interventions, as well as relevant comparisons with older interventions might be discarded, and so the full evidence-base is not utilized in the CEA. NMAs on the other hand can produce estimates of comparative efficacy for all considered options, and allow for inclusion of all relevant randomized evidence (i.e., both direct and indirect evidence). Therefore NMAs are likely to more optimally and rationally utilize the available evidence, and the resulting added precision and accuracy may translate into a more confident adoption decision.

The use of PMAs rather than NMAs for evidence synthesis in economic evaluations therefore represents a missed opportunity for optimizing decision-making [5]. To provide insights on the benefit of using NMAs, rather than PMAs, in CEAs we use an illustrative case of pharmacotherapies for chronic obstructive pulmonary disease (COPD). We demonstrate how the precision gained on efficacy estimated via the NMA, as opposed to PMA, can reduce the uncertainty around CEA outputs and can result in more confident adoption decisions. We also provide practical guidance on the step-wise processes needed to incorporate the NMA analysis into the CEA process.

## Methods and material

We use a motivating example of pharmacotherapies for the treatment of moderate to severe of COPD. COPD is a chronic disease of the airways that is responsible for a substantial economic and humanistic burden [13]. Exacerbations (lung attacks) are hallmarks of COPD, and are associated with significant costs, impaired quality of life, and risk of mortality [14]. There are multiple pharmacotherapies available for COPD and there is considerable debate on which pharmacotherapy should be used as first line treatment in COPD [15]. There is inconsistent evidence as to whether pharmacotherapies can change the course of COPD. Nevertheless, pharmacotherapies have a proven impact on reducing the exacerbation rate in COPD [16]. There are several RCTs comparing such therapies with placebo (i.e., no treatment), as well as a large number of RCTs providing head-to-head comparisons between such therapies [16].

### NMA model and data

Efficacy data was taken from a recent NMA on the effect of pharmacotherapies in reducing the exacerbation rates in patients with COPD [16]. In particular, five interventions were considered: no treatment (placebo), inhaled corticosteroids (ICS), long-acting beta-agonists (LABA), long-acting muscarinic agents (LAMA), and the combination of ICS and LABA (ICS + LABA). Several agents are available within each of these three drug classes (e.g., salmeterol, formoterol, and indacaterol are all LABAs) but they were considered equally effective in this analysis. While some may challenge this assumption, there are a number of reasons for employing this assumption in our study. First, our study is predominantly of an educational nature, and thus, simplicity in assumptions is key. Second, the NMA on which this study is based also assumed class-effects [16]. Third, other NMA that have distinguished between therapies within classes have failed to demonstrate statistically significant differences within classes [17]. Lastly, the assumption of ‘class effect’ for medications within the same class has long been an accepted paradigm in COPD [18].

**Figure 1:**
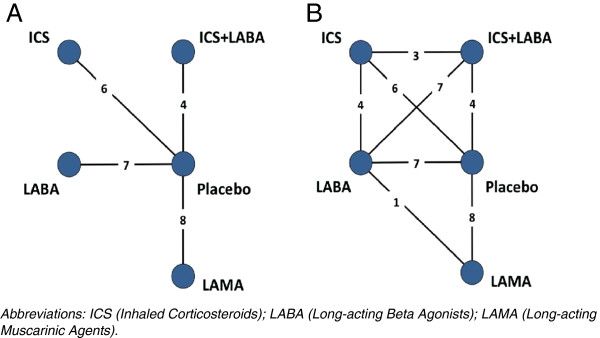
Treatment networks constituting the evidence-base used in the PMA (left) and NMA (right) analyses.

Details of the NMA are provided elsewhere [16]. The outcome (effect) of interest in synthesizing such evidence was the impact of the intervention on the yearly rate of COPD exacerbation. A total of 19 trials (14 two-arm trials, 1 three-arm trial, and 4 four-arm trials) including a total of 28,172 patients informed the evidence-base. Most interventions had been compared head-to-head in at least one RCT. The effect measure of the NMA was the rate ratio (RR) comparing each treatment versus no treatment (i.e., placebo) for yearly incidence rates of exacerbations (an RR less than one means the treatment reduced the exacerbation rate, compared with no treatment). One-year RR estimates were obtained using a Bayesian Poisson regression NMA model [10]. Separately, Bayesian Poisson regression PMAs were used to obtain conventional pair wise RRs for each of the considered interventions versus no treatment, from the placebo-based RCTs. Figure 1(A) presents the treatment network of available comparisons, and Figure 1(B) presents the full treatment network.

### Economic model and data

A decision-analytic model of COPD was created that translated the measures of treatment effect [16], combined with parameters representing the epidemiology [13,19] and natural history [20,21] of COPD, into the costs [22,23], exacerbation rates and quality-adjusted life years (QALYs) associated with each treatment [16,20,21]. The time-horizon was 10 years with one-year time cycles. A constant yearly rate of exacerbations was assumed, thus allowing for the NMA RR estimate to be employed for determining transition probabilities for each of the ten cycles. Yearly mortality rates were taken from American life Tables [24]. The yearly discount rate was set to 3% for both health and cost outcomes. The analysis adopted a third-party payer perspective. All costs were converted and presented as annual costs in year 2011 US dollars ($).

**Figure 2:**
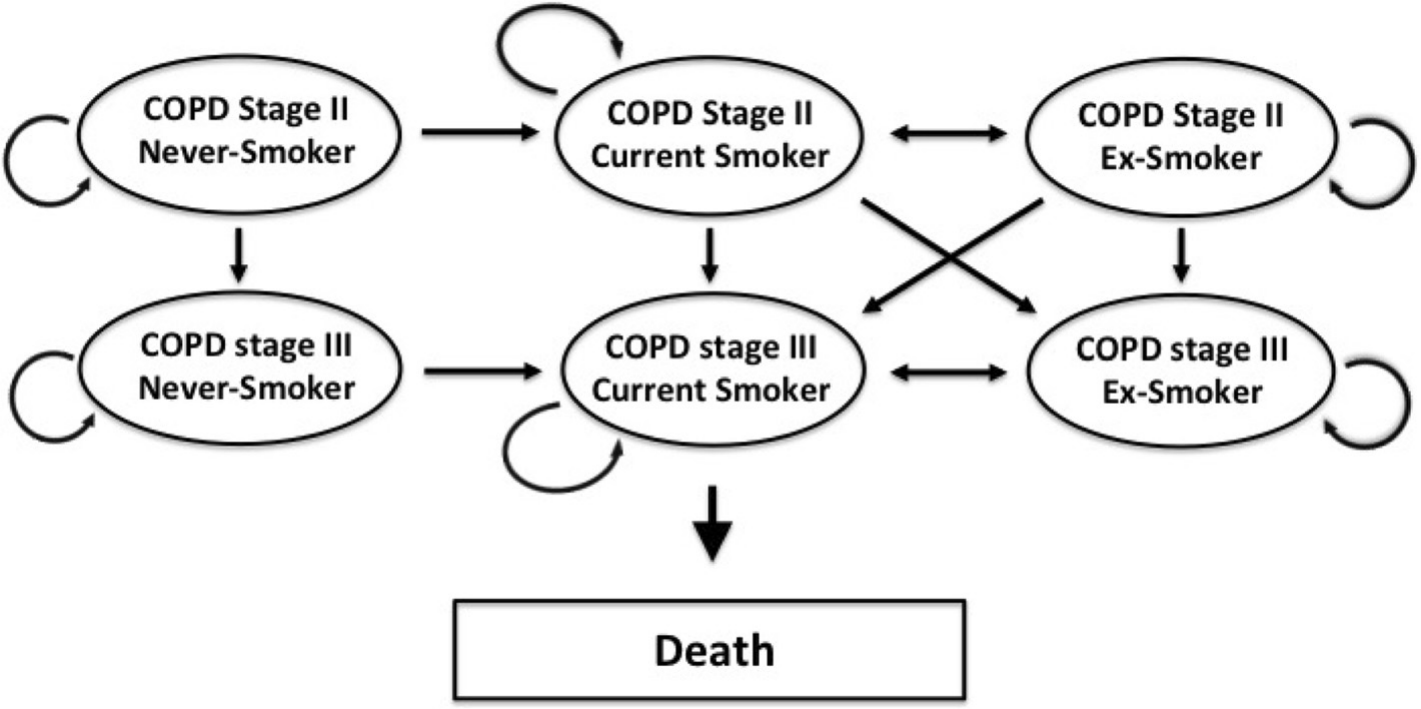
Markov model used for the performed cost-effectiveness analysis.

Figure 2 demonstrates the structure of the model. In modeling the natural history of patients with moderate to severe COPD, we used the Global Burden of Lung Disease (GOLD) criteria to classify COPD into mild, moderate, and severe. However, as the RCTs informing the evidence base evaluated the impact of treatments in patients with moderate/severe COPD, we excluded the state of mild COPD. In addition to COPD states, individuals in the model could also independently move through the states representing being a current smoker, ex-smoker, and never-smoker. Individuals could not revert from a worse COPD state to a better COPD state.

Each state of COPD was associated with an annual exacerbation rate for each treatment, which was calculated as the product of a baseline (no treatment) rate multiplied by the RR of the treatment versus no treatment. Exacerbations were categorized as either minor or major. The impact of treatment was assumed to be independent of the severity of the exacerbation.

Table 1 provides the parameter estimates and their probability distributions used to populate the model. Estimates in original reports for the majority of the parameters were accompanied by confidence intervals or standard errors. As such, each parameter was modeled as a probability distribution to match the reported level of uncertainty. On the other hand, cost components often were not accompanied by uncertainty, and we a priori decided to model costs to have a gamma distribution with a coefficient of variation of 0.25. Cost of medications were assumed fixed at their known value in 2013.

**Table 1:** Parameter estimates and their probability distributions used to populate the model

**Parameter**	**Assumed input value at GOLD stages**		**Assumed probability distribution at GOLD stages**	
	**II**	**III**	**II**	**III**
***Annual COPD mortality *** [20]	0.00393	0.006762	--	--
***Utility and disutilities *** [19]				
Baseline	0.72	0.67	B(160, 62)	B(59, 29)
Minor exacerbation	0.658	0.475	B(164, 85)	B(47, 52)
Major exacerbation	0.447	0.408	B(22, 27)	B(39, 57)
***Exacerbations rates and probabilities *** [19]				
Frequency	1.22	1.47	Γ(14884, 12200)	Γ(21609, 14700)
Minor (%)	0.93	0.90	Γ(8649, 9300)	Γ(8100, 9000)
Major (%)	0.07	0.10	Γ(12.25, 175)	Γ(25, 250)
Minor exacerbation	80$	134$	Γ(320, 4)	Γ(536, 4)
Major exacerbation	3250$	5417$	Γ(13000, 4)	Γ(21668, 4)
***Indirect maintenance cost *** [20]	215$	524$	Γ(860, 4)	Γ(2096, 4)
***Direct exacerbations costs ($) *** [19]				
Minor exacerbations	161$		Γ(644, 4)	
Major exacerbations	6501$		Γ(26004, 4)	
General practitioner visit	70$		Γ(280, 4)	
Specialist visit	90$		Γ(360, 4)	
**Direct medication costs ($) [**[22]				
Inhaled corticosteroids (ICS)	450$		--	
Long-acting beta-agonists (LABA)	500$		--	
ICS + LABA	1000$		--	
Long-acting muscarinic agents	750$		--	

### Analysis

The Bayesian NMA model was run in WinBUGS v.1.4.3 [25], and the economic model was run in R v2.14 [26]. WinBUGS and R code is available from the authors upon request. The step-wise implementation of the PMA and NMA analyses and the CEA is described further in the Additional file 1. A total of 10,000 posterior distribution samples were used for the CEA, separately for the NMA and PMA meta-analyses. The model outputs on costs and QALYs were used to calculate the ICERs and incremental net monetary benefits (INMB), with no treatment as the reference group, and to draw the cost-effectiveness planes and cost-effectiveness acceptability curves (CEACs). Treatments were also ranked according to their INMB at WTP of $50,000/QALY, separately for PMA- and NMA-based analyses.

## Results

Table 2 presents the RRs and the associated credible intervals (CrI) for all treatment vs no treatment comparisons based on the NMA and PMA meta-analyses. The pooled RR estimates for all treatment vs no treatment comparisons were similar for the NMA and PMA meta-analyses, but the NMA results had higher precision, manifested in terms of tighter CrIs (Table 2).

**Table 2:** Incidence rate ratio estimates for the considered interventions based on pair-wise meta-analysis (PMA) and network meta-analysis (NMA)

**Intervention**	**Rate ratios (95% CrI)**	
	**PMA**	**NMA**
Placebo		
ICS	0.81 (0.68-0.95)	0.81 (0.72-0.91)
LABA	0.87 (0.75-1.01)	0.87 (0.78-0.96)
ICS + LABA	0.71 (0.60-0.88)	0.70 (0.62-0.79)
LAMA	0.73 (0.59-0.91)	0.74 (0.67-0.82)

PTC: pairwise treatment comparison, MTC: multiple treatment comparison, CrI: credible interval.

**Figure 3:**
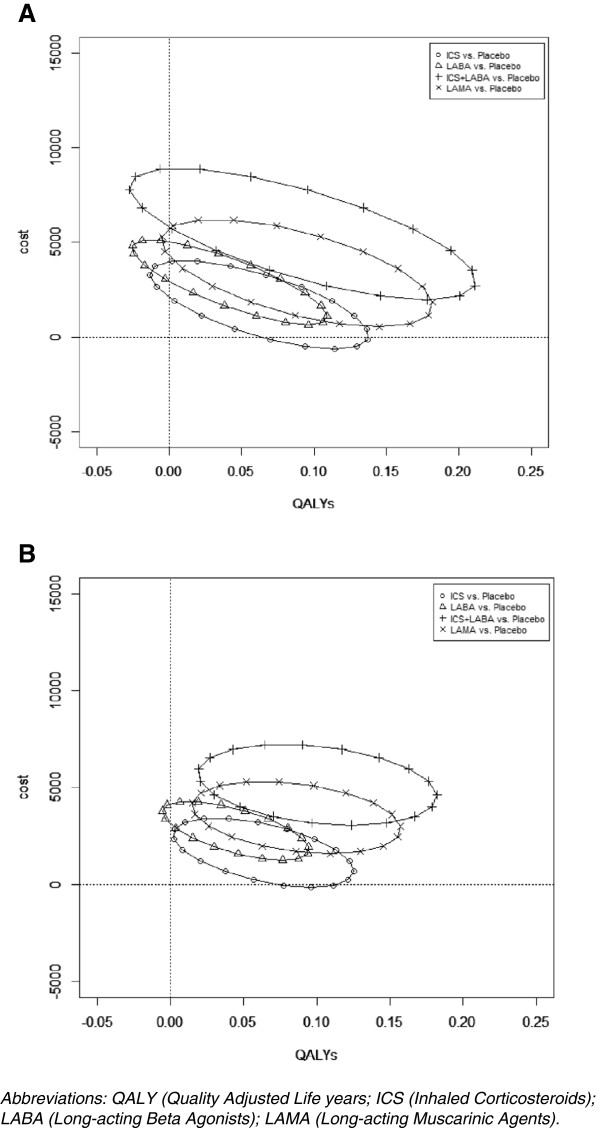
Cost-effectiveness plane illustrating the 95% credible ellipses for each intervention versus placebo based on the pair wise meta-analysis (A) and the network meta-analysis (B).

Table 3 presents the mean and 95% CrIs for costs, exacerbation rates, and QALYs. Figure 3 presents the uncertainty ellipses around the incremental cost and QALY estimates on the cost-effectiveness plane. Uncertainty around both costs and QALYs was reduced substantially in the NMA-based analysis. This reduction is visually apparent from the considerably smaller 95% credible ellipses NMA-based analysis compared with the PMA-based analysis in Figure 3.

**Table 3:** 10-year average cost, number of exacerbations, and quality adjusted life-years for each intervention using both pairwise meta-analysis (PMA) and network meta-analysis (NMA)

**Intervention**	**Meta-analysis**	**Costs ($)**	**Number of exacerbations**	**Quality adjusted life years (QALYs)**
Placebo	PMA	25 458 (19 927, 32 312)	12.6 (12.5, 12.8)	5.67 (5.36, 5.99)
	NMA	25 316 (19 950, 31 897)	12.6 (12.5, 12.7)	5.67 (5.34, 5.99)
ICS	PMA	27 116 (22 194, 33 307)	10.3 (8.62, 11.9)	5.73 (5.40, 6.07)
	NMA	26 979 (21 991, 33 420)	10.2 (9.10, 11.4)	5.73 (5.38, 6.06)
LABA	PMA	28 304 (23 081, 34 897)	11.0 (9.31, 13.2)	5.71 (5.39, 6.04)
	NMA	28 116 (23 002, 34 604)	10.9 (9.86, 12.2)	5.71 (5.37, 6.05)
ICS + LABA	PMA	30 849 (25 947, 36 741)	9.13 (7.69, 11.2)	5.76 (5.42, 6.10)
	NMA	30 496 (25 902, 36 133)	8.85 (7.83, 10.0)	5.75 (5.40, 6.11)
LAMA	PMA	28 840 (23 928, 35 073)	9.33 (7.47, 11.4)	5.76 (5.42, 6.10)
	NMA	28 816 (24 237, 34 548)	9.39 (8.37, 10.4)	5.77 (5.41, 6.12)

**Figure 4:**
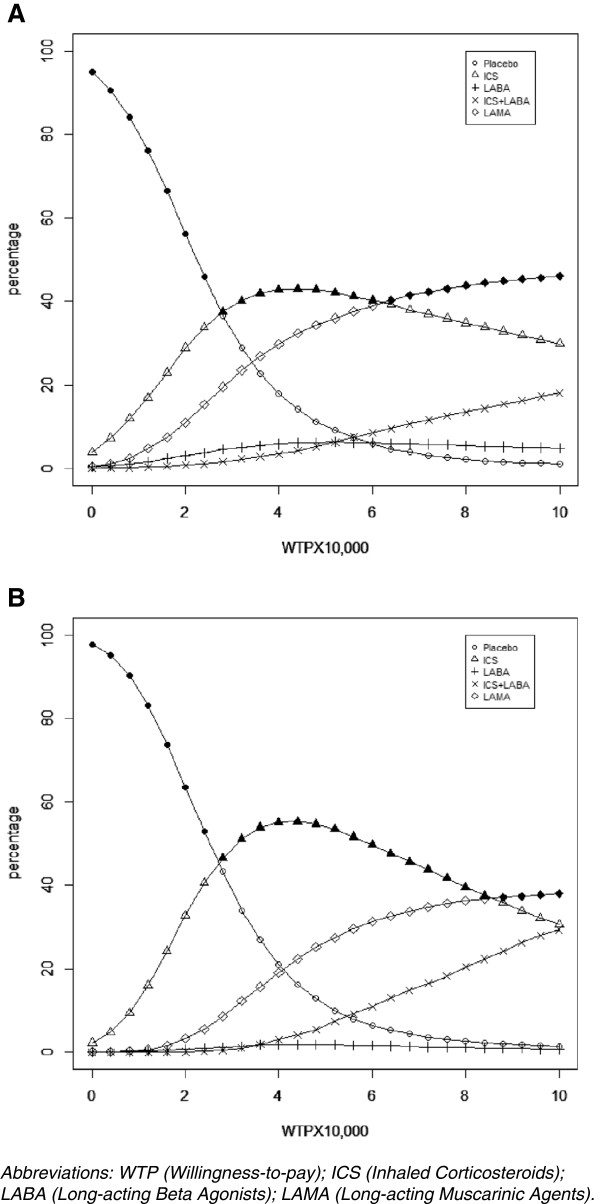
Cost-effectiveness acceptability curves when efficacy results are based on the pair wise meta-analysis (A) and the network meta-analysis (B).

Table 4 presents the ICERs and probabilities of each treatment being cost-effective as WTP thresholds of $30,000, $50,000, $70,000, and $100,000. Figure 4 presents the CEACs for all interventions from WTP thresholds between $0/QALY and $100,000/QALY. The ICERs from the PMA- and NMA-based analyses were similar, but the probabilities of being cost-effective at the explored WTP thresholds varied considerably. The largest difference in the probabilities of being cost-effective was observed at a WTP of approximately $40,000/QALY. At this threshold, with the PMA-based analysis, ICS, LAMA and placebo had a 43%, 30%, and 18% probability of being the most cost-effective. By contrast, with the NMA based approach, ICS, LAMA, and placebo had a 56%, 19%, and 21% probability of being cost-effective. As illustrated in both Table 4 and Figure 4, the differences between the two approaches were also notable for all WTP thresholds above approximately $25,000. In both analyses, LAMA were estimated more likely to be cost-effective than ICS for high WTP threshold, but the point where these probabilities crossed were different between the PMA- and NMA-based analyses. In particular, with the PMA-based approach the point of probabilities crossing was between $60,000/QALY and $65,000/QALY, whereas the point of crossing with the NMA-based approach was between $85,000/QALY and $90,000/QALY.

**Table 4:** Incremental cost-effectiveness ratios (ICERs) and probabilities of each intervention being the most cost effective at various willingness-to-pay thresholds

**Intervention**	**Meta-analysis**	**ICER**	**Probability of being cost-effective by willingness-to-pay threshold**			
			**$30,000/QALY**	**$50,000/QALY**	**$70,000/QALY**	**$100,000/QALY**
***Placebo as reference***						
Placebo	PTC	Reference	35%	10%	3%	0%
	MTC	Reference	37%	12%	3%	2%
ICS	PTC	27044	40%	42%	38%	31%
	MTC	27614	49%	55%	44%	31%
LABA	PTC	65509	1%	6%	6%	5%
	MTC	64339	1%	2%	1%	0%
ICS + LABA	PTC	57933	1%	6%	11%	18%
	MTC	52116	0%	7%	1%	29%
LAMA	PTC	38427	21%	34%	43%	46%
	MTC	41203	13%	24%	35%	38%
***ICS as reference***						
LABA	PTC	Dominated	--	--	--	--
	MTC	Dominated	--	--	--	--
ICS + LABA	PTC	89843	--	--	--	--
	MTC	96749	--	--	--	--
LAMA	PTC	25930	--	--	--	--
	MTC	57854	--	--	--	--

At WTP of $50,000/QALY, the ranking of the first three treatments (ICS, LAMA, and no treatment) remained the same between PMA- and NMA-based analyses. The only difference in the results was that the treatment with the lowest INMB for the PMA-based analysis was ICS + LABA whereas for the NMA-based analysis it was LABA.

## Discussion

In the present work we elaborated on the theoretical advantages of using NMAs over PMAs in economic evaluations of health technologies, and used a case study to demonstrate the practical aspects of the use of NMAs as well as the empirical differences in the outcomes of the economic evaluation when NMA instead of PMA is used for evidence synthesis. The results demonstrate how the CEA can benefit from the gain in precision from using the entire network of evidence rather than the results of pair wise comparisons alone. In our case study, while the added precision did not result in major changes in the choice of the optimal treatment across a wide range of WTP, it prevented the counter-intuitive situation of the optimal treatment not having the maximum probability of cost-effectiveness [27].

The network of evidence underlying the case study was a well-connected treatment network including large studies and head-to-head RCTs for almost all comparisons. As such, the use of the entire available evidence base synthesized through NMA resulted in similar point estimate for the effect size but with an increased precision. However, situations may occur where NMA estimates are not close to their PMA counterparts; and where the combination of indirect and direct evidence does little to increase the precision [7]. However, the theoretical justifications underpinning the use of NMA instead of PMA are unrelated to the empirical gains in certainty and stand valid regardless of any particular results.

The performed analyses come with some limitations. We used a simple decision-analytic model of COPD for the case study, mainly based on the modeling assumptions used by previous authors [20,21]. The simplicity of this model allowed us to focus on the practical aspects and illustration of the results; but we acknowledge that to inform policy, a deeper analysis, including a detailed set of sensitivity and alternative analyses will be required. For example, our model did not account for the potential impact of treatments on disease progression [20], a controversial aspect of the treatment that needs to be considered in a sensitivity analysis. Our model also did not account for potential long-term adverse events associated with corticosteroid treatment and their associated costs. However, the complexity of building a decision-model is not intensified by the use of NMA versus PMA for evidence synthesis.

The implications of the results are rather straightforward: the potential theoretical and practical gains in using NMAs as opposed to PMAs in cost-effectiveness analysis are too significant to be ignored. However, this does not mean that CEAs should only ever rely on efficacy estimates from NMAs. NMA is a method of inference and as such is based on certain statistical assumptions that are generally more restrictive than the assumptions underlying PMA [2,3]. For example, there are situations where NMA estimates may be more biased than their PMA counterparts estimated only from placebo comparisons [28,29]. If in a particular context where there are misgivings about the suitability of such assumptions, the investigator might deliberately choose PMA. Overall, a thorough assessment of the potential biases and confounders in both the NMA and the PMA is necessary before deciding which data and type of research synthesis method should be used for informing the cost-effectiveness analysis.

## Conclusion

In summary, incorporating NMA in CEA offers consistency and added certainty in comparison with CEA informed by conventional PMA. As the role of NMAs in informing comparative efficacy in the evaluation of new health technologies is growing, NMAs could and should be considered for informing the evidence used in CEA.

## Competing interest

Kristian Thorlund and Edward Mills are founding partners of Redwood Outcomes Inc. Redwood Outcomes consults to a number pharmaceutical companies, of which several are manufacturers of at least one brand belonging to the classes of COPD drugs considered for this article.

## Authors’ contributions

KT conceived the idea of the study, contributed to the design of the study, wrote up the first manuscript, conducted the pair wise meta-analysis and network meta-analysis, and contributed to the interpretation of findings. ZZ contributed to the design of the study, programmed the Markov model and ran all cost-effectiveness analyses, contributed to the writing of the manuscript and contributed to the interpretation of findings. ED contributed to the design of the study, contributed to the identification of literature to inform model parameter values, contributed to the writing of the manuscript and contributed to the interpretation of findings. EM contributed to the design of the study, the writing of the manuscript, and the interpretation of findings. MS contributed to the design of the study, contributed to the identification of literature to inform model parameter values, supervised the development of the cost-effectiveness Markov model, contributed to the writing of the manuscript and contributed to the interpretation of findings. All authors read and approve the final manuscript.

## Supplementary Material

Additional file 1 Implemention of WinBUGS and R. Click here for file
