# Supplementary material for: The impact of incorporating Bayesian network meta-analysis in cost-effectiveness analysis - a case study of pharmacotherapies for moderate to severe COPD
Source: Cost Eff Resour Alloc. 2014 Mar 13;12:8. doi: 10.1186/1478-7547-12-8 (PMC4007707; doi:10.1186/1478-7547-12-8)
Supplement: Additional file 1 — Implemention of WinBUGS and R. [file 1478-7547-12-8-S1.docx]

**Additional file 1**

**Appendix 1 - Implemention of WinBUGS and R**

WinBUGS and R were ‘merged’ by calling WinBUGS from R using the *BRugs* package. In particular, this process consisted of the following steps: 1) R called WinBUGS and WinBUGS ran the Bayesian MTC analysis; 2) R recorded a pre-specified number of paired MCMC samples (after burn-in) from the posterior distributions of the rate ratios for each intervention versus placebo; 3) each of the sampled RRs were converted into expected incidence rates for each intervention group using some assumed placebo yearly incidence rate; 4) the obtained sample of intervention group incidence rates were fed into the economic model, which was run and setup to output the desired health economic outputs (e.g., cost effectiveness plane). The same process applied to PTC meta-analysis, with the exception that WinBUGS needs to be called multiple times from R for each placebo comparison pair wise meta-analysis relevant to the economic analysis
